# Ultrasound diagnosis of acrania with major low–lying placenta and polyhydramnios; case report

**DOI:** 10.4314/gmj.v55i2.12

**Published:** 2021-06

**Authors:** Jared N Oblitey, William K Antwi, Benard O Botwe, Mary K Oblitey

**Affiliations:** 1 Department of Radiography, School of Biomedical and Allied Health Sciences, University of Ghana, Korle Bu, Accra, Ghana; 2 Department of Radiology, Korle Bu Teaching Hospital, Accra, Ghana

**Keywords:** Acrania, exencephaly, anencephaly, major low-lying placenta, placental cyst

## Abstract

Acrania is a rare foetal anomaly in which the calvaria is absent, and the meninges come into direct contact with the amniotic fluid. Acrania is the most common anomaly in the acrania – exencephaly – anencephaly spectrum, with an incidence of 3.68 to 5.4 per 10,000 live births. We present a case of a primigravida who presented for an ultrasound on account of vaginal bleeding in early cyesis. Transabdominal ultrasound showed a viable foetus at 13 weeks without a calvaria, with the brain in direct contact with amniotic fluid. There was a low-lying placenta extending from the posterior to anterior part of the lower uterine segment, completely covering the internal cervical os (major low–lying placenta), a placental cyst and polyhydramnios (amniotic fluid index, AFI of 17 cm). A diagnosis of acrania with major low–lying placenta and polyhydramnios was made. Detailed ultrasound is required to detect acrania at 13 weeks. The diagnosis of acrania is required to help direct patient counselling and maternal expectation. When acrania and major low–lying placenta occur in the same patient, both diagnoses must be promptly made concurrently, regardless of gestational age and without waiting for placental trophotropism and migration to occur first.

**Funding:**

None declared

## Introduction

Acrania is a foetal anomaly in which there is a complete or partial absence of the calvaria above the orbits and supraciliary ridge, allowing the meninges to come into direct contact with amniotic fluid. Acrania is the most common anomaly in the acrania–exencephaly–anencephaly spectrum, with an estimated incidence ranging from 3.68 to 5.4 per 10,000 live births.^1^ When acrania occurs, the brain is exposed to the amniotic fluid with a risk of mechanical and chemical trauma through friction with the uterine wall, placenta and foetal parts.^2^ Subsequently, the brain may suffer progressive degenerations that could ultimately lead to partial or total disappearance of the brain tissues, particularly from the 14th week of gestation.^2^,^3^ This presentation seeks to highlight a case of acrania diagnosed at 13 weeks to raise the index of suspicion among obstetricians and radiologists.

## Case Report

A primigravida presented at a health facility for a firsttrimester ultrasound examination. She had been experiencing bleeding *per vaginam* with blood clots, and her obstetricians were concerned she might have been aborting. There was no relevant past obstetric history.

**Figure 1:**
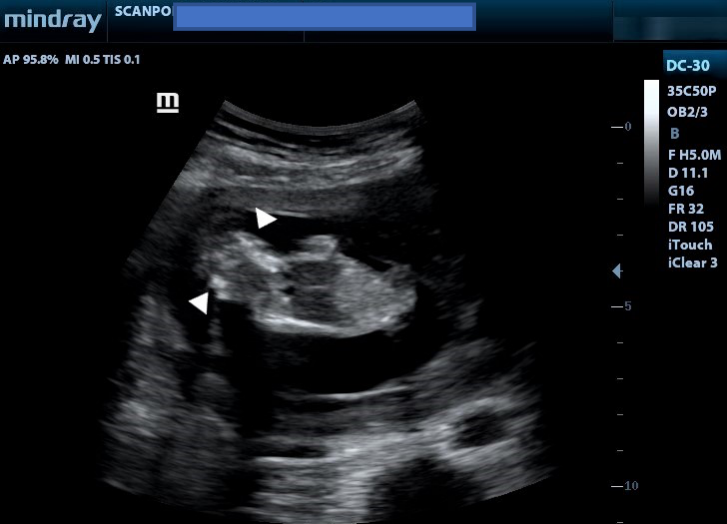
Coronal Ultrasound image showing a foetus at 13 weeks with no calvaria. White arrowheads show the brain tissue without bony covering. The amniotic fluid index (AFI) was 17 cm

**Figure 2:**
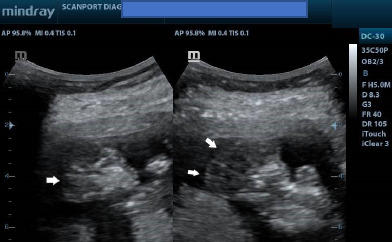
Ultrasound images showing sagittal and parasagittal images of the brain demonstrating the absence of the calvaria. See the margins of the brain (white arrows) without bony margins

**Figure 3:**
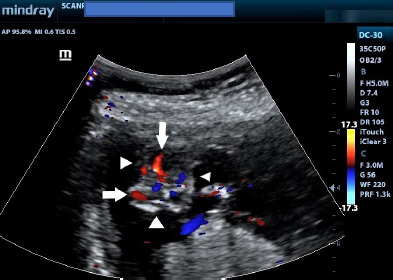
Axial Colour Doppler ultrasound images of the head showing the absence of the calvaria. Note the dysplastic vessels (white arrows) within the brain. The arrowheads indicate the margins of the bare brain

Ultrasound showed a viable foetus at 13 weeks gestation (Figure 1) with increased amniotic fluid (amniotic fluid index, AFI of 17cm). The brain appeared well-formed, but the bony landmarks for the biparietal diameter (BPD) measurement were difficult to obtain. In addition, the brain had no bony covering (Figure 2) and appeared to bulge freely into the amniotic fluid, with dysplastic intracerebral vessels on colour Doppler (Figure 3).

**Figure 4:**
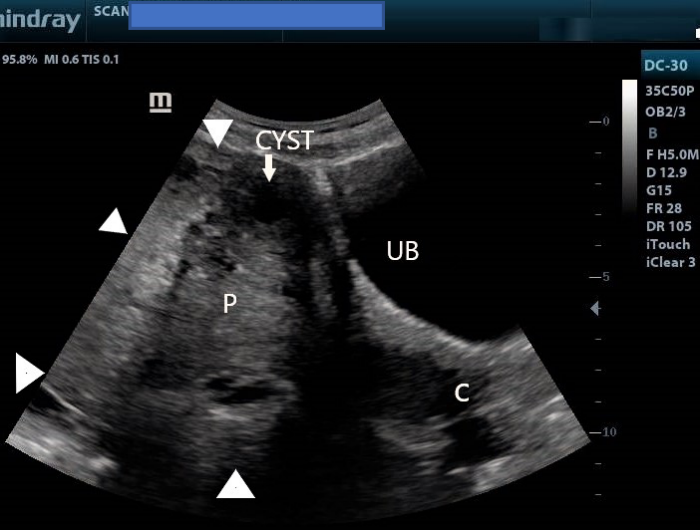
Lower uterine segment ultrasound image showing the urinary bladder and internal cervical os (C) completely covered by the placenta from anterior to posterior. White arrowheads indicate placental margins. Note also the anterior placental cyst. UB refers to the urinary bladder

**Figure 5:**
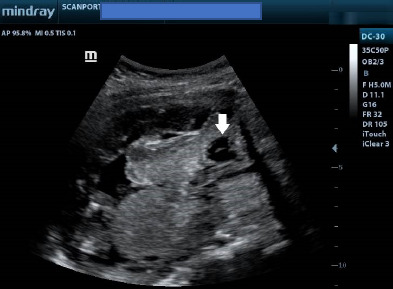
Ultrasound image of the lower uterine segment showing the placenta completely covering the internal cervical os. Note also the cystic lesion (white arrow) at the anterior part of the placenta, in keeping with a placental cyst

The placenta completely covered the internal cervical os (Figure 4 and Figure 5). In addition, there was an anechoic cyst with an internal septum at the anterior part of the placenta, suggesting a placental cyst (Figure 5). A diagnosis of acrania with major low–lying placenta, placental cyst and polyhydramnios was made. The patient was referred back to an obstetrician for counselling and continued management. Consent for the case to be published (including images, case history and data) was obtained from the patient.

## Discussion

The acrania – exencephaly – anencephaly spectrum sequence is the most severe neural tube defect and results from a failure to close the rostral end of the neural tube and abnormal migration of mesenchymal tissue which normally covers the cerebral hemispheres.^4^ Embryologically, this failure of migration occurs at the beginning of the 4th week, and the major insult is a lack of cranial development.

The main mode of diagnosing acrania is by obstetric ultrasound.^5^ The calvaria was absent in the foetus in this case. The International Society of Ultrasound and Gynaecology (ISUOG) has issued guidelines emphasizing the need to recognise the foetal head, cranial bones, choroidplexus and cerebral ventricles for all first trimester scans.^6^ According to Santana et al. 6, from the 11^th^ week onwards, cranial ossification ought to be evident. It was, however, reported in Denmark^7^ that detection rates for the acrania- exencephaly – anencephaly were low on basic scans done before the 11^th^ week, requiring expert maternal – foetal sonologists for improved detection.^7^

The key sonographic features of acrania are absent calvaria. The damaged brain tissue presents as a ‘Mickey Mouse’ sign^3^ in the early stages and then as echogenic particles in the amniotic fluid in advanced stages of the condition.

These echogenic particles lead to an increased amniotic fluid texture, a feature reported by both Cafici^5^ and Santana^6^ as a likely very first indication of the spectrum. It has thus been suggested that this feature could be a potential marker for first-trimester acrania. However, such echogenic particles were not noted in this current case, likely because much brain destruction had not yet occurred at the time of examination.

It has also been suggested that the acrania – exencephaly – anencephaly spectrum is actually the same disorder but presents as acrania in the first trimester and later as anencephaly after the brain destruction has occurred.^5^ For instance, during one longitudinal study in the same cohort of patients^8^, it was noted that exencephaly was the predominant finding in the first trimester. In contrast, in the second trimester, the classic appearance of anencephaly was seen more often. In fact, in one particular foetus, the transition from exencephaly to anencephaly was documented by serial ultrasound exams.^8^

Many types of the acrania–anencephaly spectrum have been described. One retrospective study of 88 cases of the spectrum concluded that there are six subtypes; described as overhanging, elongated, bilobular, cystic, foreshortened, and irregular, with the first three accounting for 75% of all cases.^9^ In his pictorial essay, Santana et al^1^ showed images of acrania with cysts in the brain, describing them as cystic acrania. Sepulveda et al^10^ also recently described a previously unknown firsttrimester presentation of the sequence in three foetuses. There was a constriction ring noted around the external skull base with an enlarged globular head. It was, therefore, suggested that this subtype of the sequence may be due to segmental amniotic rupture with the remnants entrapping the foetal head. All three cases were either aborted therapeutically or suffered intrauterine demise.

The current case was associated with polyhydramnios (AFI of 17cm; reference range at 15 weeks – 8.7 -13.7 cm, 5^th^ -95^th^ percentile)^11^, a feature also reported by Santana.^1^ In Cameroun, Koaum^12^ described a case of anencephaly complicated by acute polyhydramnios and underscored the need to actively screen for anomalies in all cases of polyhydramnios.

This current case showed acrania with major low–lying placenta diagnosed at 13 weeks and a cystic area within the placenta. Oppenheimer^13^ also showed images of a patient with placenta percreta, placenta praevia and anencephaly. However, whether a relationship exists between the acrania–exencephaly–anencephaly spectrum and low–lying placenta is not clear.

Oppenheimer^13^ further asserted that because of normal trophotropism and first-trimester migration of the placenta, a diagnosis of placenta praevia should not be made until after 15 weeks gestation. However, there may be spontaneous abortion or elective termination in cases of acrania shortly after diagnosis and before placental migration has occurred. Therefore, the early diagnosis of placenta praevia becomes critical to help craft a delivery plan that prevents maternal haemorrhage.^14^ In our opinion, therefore, whenever acrania and major low–lying placenta occur in the same patient, the diagnosis of major placenta praevia must be made promptly, regardless of gestational age and without waiting for placental migration or trophotropism (which may not be allowed to occur in such a patient). We further assert that such a clear diagnosis of Major placenta praevia (even when made in the early second trimester) helps quickly classify the patient as high risk and appropriate interventions instituted. The differential diagnoses of acrania include osteogenesis imperfecta and hypophosphatasia. In both cases, however, bones are present around the brain but are poorly mineralized. The presence of other bones with fractures may also help distinguish acrania from osteogenesis imperfecta.^15^

Management of acrania is generally by elective termination. However, in all cases, early in - utero diagnosis helps manage maternal expectations and directs appropriate counselling. In most cases, the prognosis is lethal, with 65% dying in –utero and 35% dying during delivery. Short term survival (minutes to days) has also been reported as well as a 2 – 5% recurrence risk in future pregnancies.^16^

## Conclusion

Detailed ultrasound is required to detect acrania at 13 weeks gestation. The diagnosis of acrania is required to help direct patient counselling and maternal expectation. When acrania and major low–lying placenta occur in the same patient, both diagnoses must be made promptly and concurrently regardless of gestational age and without waiting for placental trophotropism and placental migration to occur first.
